# A Case of Healthcare Associated Pneumonia Caused by *Chryseobacterium indologenes* in an Immunocompetent Patient

**DOI:** 10.1155/2015/483923

**Published:** 2015-01-06

**Authors:** Salih Atakan Nemli, Tuna Demirdal, Serap Ural

**Affiliations:** Department of Infectious Diseases, Ataturk Training and Research Hospital, Izmir Katip Celebi University, 35360 Izmir, Turkey

## Abstract

*Chryseobacterium indologenes* is nonmotile, oxidase, and indole positive Gram-negative aerobic bacilli which is widely found in plants, soil, foodstuffs, and water. It can colonize hospital environment due to ability to survive in chlorine-treated water supplies. Chryseobacteria can also colonize patients via contaminated medical devices such as respirators, intubation tubes, humidifiers, intravascular catheters, and prosthetic valves. Immune suppression, comorbidities, use of broad-spectrum antibiotics, and extreme age are other important risk factors for *Chryseobacterium* infections. We report a case of an 82-year-old male admitted to our hospital with the complaint of altered mental status with history of trauma, and recent orthopedic and neurosurgery operations. He was transferred to neurosurgery intensive care unit due to respiratory failure. Urine culture yielded extended spectrum beta lactamase (ESBL) (+). *E. coli* and *C. indologenes* were isolated from transtracheal aspirate. He was treated with ertapenem, and levofloxacin and discharged with full recovery.

## 1. Introduction

*Chryseobacterium indologenes* formally known as* Flavobacterium indologenes* is nonmotile, oxidase, and indole positive Gram-negative aerobic bacilli. It is widely found in plants, soil, foodstuffs, and water [1]. It has ability to survive in chlorine-treated municipal water supplies and can colonize sink basins and taps. Thus, it can easily colonize hospital environment and can be a potential source for healthcare associated infections (HAIs) [2]. Chryseobacteria can also colonize patients via contaminated medical devices such as respirators, intubation tubes, humidifiers, intravascular catheters, and prosthetic valves [3, 4]. Though* Chryseobacterium species* are infrequent human pathogen, it can be identified as causative agent in patients with pneumonia [5], bacteremia [6], sepsis [7], urinary tract infection [8], peritonitis [9], and ocular infection [10]. Patients with long-term indwelling devices and prolonged exposure to broad-spectrum antibiotics are at high risk for* Chryseobacterium indologenes* infections. Increased use of colistin and tigecycline against multidrug resistant bacteria such as* Acinetobacter baumannii* is associated with increased prevalence of* Chryseobacterium* infections in intensive care units [11].

## 2. Case Report

82-year-old male patient was admitted to hospital with the complaint of altered mental status in July 2014. In April 2014, he was taken to hospital because of traffic accident. Computerized tomography (CT) was performed and it revealed subarachnoid hemorrhage. He was followed up at neurosurgery clinic. He was operated on because of left tibia fracture and discharged with full recovery two weeks later. He was admitted to neurosurgery clinic again with the complaints of vertigo, nausea, and vomiting in June 2014. CT revealed chronic subdural hematoma and subdural hygroma. At the same day, he was immediately operated on and haematoma was evacuated. He was discharged ten days later. Then he was admitted to neurosurgery clinic again with altered mental status in June 23, 2014. Physical examination revealed no pathological findings except disorientation. Laboratory tests revealed leucocyte 7.8 K/*μ*L, hemoglobin 10.8 g/dL, platelet 252 K/*μ*L, procalcitonin 0.26 ng/mL, and CRP 8.18 mg/dL. Urine tests revealed pyuria and urine culture was performed. Brain CT was performed and bilateral subdural hematoma was noted. On the second day of admission dyspnea occurred, and he was intubated and transferred to neurosurgery intensive care unit. Thorax CT was performed; an air cyst in right lobe, bronchiectasis, and a consolidated area in left lobe were noted. Piperacillin/tazobactam therapy was initiated empirically. On the third day, transtracheal aspirate sample was sent to microbiology laboratory. Antimicrobial therapy was modified to ertapenem after urine culture yielded ESBL (+).

*E. coli* and* C. indologenes* were isolated from transtracheal aspirate sample. Antimicrobial susceptibility test revealed resistance to carbapenems and sensitivity to levofloxacin. Levofloxacin was added to antimicrobial therapy on the 6th day of admission. On follow-up, after the second day of therapy modification, improvement on blood gas parameters was observed and the patient was extubated. He was transferred to neurosurgery clinic and antimicrobial therapy continued. He was discharged on the 15th day of admission with cure.

## 3. Discussion

*Chryseobacterium species* are usually nosocomial pathogens and are associated with invasive device utilization such as vascular catheters and endotracheal tubes [4, 12]. Immune suppression, comorbidities, use of broad-spectrum antibiotics, and extreme age are other important risk factors for* Chryseobacterium* infections [13].* C. indologenes* has not been frequently recovered from clinical specimens but infections have been associated with a high mortality rate [14]. Antimicrobial susceptibility pattern is not well defined because it is a rare pathogen isolated from clinical specimens. Production of class A b-lactamase and class B carbapenem-hydrolyzing b-lactamase molecules causes intrinsic carbapenem and cephalosporin resistance. It is usually susceptible to levofloxacin, trimethoprim-sulfamethoxazole, and piperacillin-tazobactam. Ciprofloxacin, cefepime, and ceftazidime have also high activity against* C. indologenes*. Therefore, it is usually resistant to aminoglycosides, other b-lactams, chloramphenicol, linezolid, and glycopeptides [6, 15–17].

SENTRY Program [2] during the 5-year period 1997 to 2001 revealed that* Chryseobacterium* species constitute only 0.03% of all bacterial isolates. All strains came from hospitalized patients.* C. meningosepticum* was the most frequently isolated microorganism followed by* C. indologenes*. Lower respiratory tract (52%) and blood (46%) were the major sites microorganism isolated. One-half of the isolates from the respiratory tract were* C. indologenes* and 42.3% were* C. meningosepticum*. The highest frequency of* Chryseobacterium* spp. infection occurred among the patients over 65 years old.

The largest case series including 91 pneumonia and 22 bacteremia patients is reported by Chen et al. [11] from Taiwan. Mechanical ventilation, use of corticosteroids, malignancy, chronic renal disease, hypertensive cardiovascular disease, diabetes, and tracheostomy were the most frequent underlying medical conditions in patients. Almost 40% of patients had history of previous carbapenem, quinolone, or broad-spectrum cephalosporin use. They also reported a correlation between* C. indologenes* isolation and increasing consumption of colistin or tigecycline. Mortality was 35% for pneumonia and 64% for bacteremia.

The vast majority of reports of* C. indologenes* infections have been published as case reports. Immunosuppression, older age, prolonged antibiotic use, and trauma were the most common clinical features reported in the majority of case reports [6, 12, 15, 16, 18–21].

The current case had no immunocompromising situation but risk factors such as history of trauma, surgery, and use of broad-spectrum antibiotics were defined as above.

## 4. Conclusion

Though* C. indologenes* infection is a rare nosocomial pathogen especially among immunocompromised patients, our case report demonstrates that this pathogen should be considered in patients with selected situations.
